# The transcription factor CgHaa1 plays a role in virulence of the pathogenic yeast *Candida glabrata*

**DOI:** 10.1093/femsyr/foaf054

**Published:** 2025-09-18

**Authors:** Sara Barbosa Salazar, Nuno Alexandre Pedro, Sónia Silva, Dalila Mil-Homens, Andreia Pimenta, Marcin Wlodarczyk, Aleksandra Szwed-Georgiou, Kaname Sasamoto, Hiroji Chibana, Sylwia Michlewska, Karolina Rudnicka, Arsénio Fialho, Nuno Pereira Mira

**Affiliations:** iBB, Institute for Bioengineering and Biosciences, Instituto Superior Técnico – Department of Bioengineering, Universidade de Lisboa, Av. Rovisco Pais, 1049-001 Lisboa, Portugal; Associate Laboratory i4HB—Institute for Health and Bioeconomy, Instituto Superior Técnico, Universidade de Lisboa, Av. Rovisco Pais, 1049-001 Lisboa, Portugal; iBB, Institute for Bioengineering and Biosciences, Instituto Superior Técnico – Department of Bioengineering, Universidade de Lisboa, Av. Rovisco Pais, 1049-001 Lisboa, Portugal; Associate Laboratory i4HB—Institute for Health and Bioeconomy, Instituto Superior Técnico, Universidade de Lisboa, Av. Rovisco Pais, 1049-001 Lisboa, Portugal; CEB - Centre of Biological Engineering, University of Minho, Campus de Gualtar, 4710-057 Braga, Portugal; LABBELS - Associate Laboratory, 4710-057 Braga, Guimarães, Portugal; iBB, Institute for Bioengineering and Biosciences, Instituto Superior Técnico – Department of Bioengineering, Universidade de Lisboa, Av. Rovisco Pais, 1049-001 Lisboa, Portugal; Associate Laboratory i4HB—Institute for Health and Bioeconomy, Instituto Superior Técnico, Universidade de Lisboa, Av. Rovisco Pais, 1049-001 Lisboa, Portugal; iBB, Institute for Bioengineering and Biosciences, Instituto Superior Técnico – Department of Bioengineering, Universidade de Lisboa, Av. Rovisco Pais, 1049-001 Lisboa, Portugal; Associate Laboratory i4HB—Institute for Health and Bioeconomy, Instituto Superior Técnico, Universidade de Lisboa, Av. Rovisco Pais, 1049-001 Lisboa, Portugal; Department of Immunology and Infectious Biology, Faculty of Biology and Environmental Protection, University of Lodz, 90-237 Łódź, Poland; Department of Immunology and Infectious Biology, Faculty of Biology and Environmental Protection, University of Lodz, 90-237 Łódź, Poland; Medical Mycology Research Center, Chiba University, Chiba 260-8673, Japan; Medical Mycology Research Center, Chiba University, Chiba 260-8673, Japan; Laboratory of Microscopic Imaging and Specialized Biological Techniques, Faculty of Biology and Environmental Protection, University of Lodz, 90-237 Łódź, Poland; Department of Immunology and Infectious Biology, Faculty of Biology and Environmental Protection, University of Lodz, 90-237 Łódź, Poland; iBB, Institute for Bioengineering and Biosciences, Instituto Superior Técnico – Department of Bioengineering, Universidade de Lisboa, Av. Rovisco Pais, 1049-001 Lisboa, Portugal; Associate Laboratory i4HB—Institute for Health and Bioeconomy, Instituto Superior Técnico, Universidade de Lisboa, Av. Rovisco Pais, 1049-001 Lisboa, Portugal; iBB, Institute for Bioengineering and Biosciences, Instituto Superior Técnico – Department of Bioengineering, Universidade de Lisboa, Av. Rovisco Pais, 1049-001 Lisboa, Portugal; Associate Laboratory i4HB—Institute for Health and Bioeconomy, Instituto Superior Técnico, Universidade de Lisboa, Av. Rovisco Pais, 1049-001 Lisboa, Portugal

**Keywords:** CgHaa1, *Candida glabrata* interaction with immune cells, adherence to vaginal cells, acetate/acetic acid

## Abstract

*Candida glabrata* is a prominent causative agent of mucosal and disseminated human infections. Part of the success of *C. glabrata* as a human pathogen relies on its adherence capacity and ability to tolerate/surpass the activity of immune cells. Herein we describe the involvement of the transcription factor CgHaa1 and of its regulated genes *CgAWP12, CgAWP13, CAGL0H07469 g*, and *CAGL0K10164 g* in adherence of *C. glabrata* to vaginal cells in the presence of acetic acid, an organic acid usually found in this niche due to the activity of commensal bacteria. CgHaa1 and its target genes *CgAWP12, CAGL0K10164 g* and *CAGL0E03740 g* were also found to significantly increase *C. glabrata*-induced killing of the model wax moth *Galleria mellonela*, in part by modulating the interaction of the yeasts with the larvae’s immune cells. Finally, we show that *CgHAA1* expression reduces ingestion and subsequent killing of *C. glabrata* cells by THP-1 human macrophages. This demonstrated role of CgHaa1 in *C. glabrata* virulence and interaction with immune cells expands the biological role of this regulator positioning it (and its target genes) as a potential interesting candidate target for new therapies focused on reducing the burden of candidiasis.

## Teaser

The transcriptional regulator CgHaa1 (and some of its target genes) contributes to the ability of *Candida glabrata* to colonize vaginal cells in the presence of acetic acid and augments virulence of the yeast.

## Introduction

*Candida glabrata* is a pathogenic yeast recognized for its ability to cause infections with a wide spectrum of severity, ranging from mild rashes in the oral or genitourinary mucosa to deadly disseminated mycoses [as reviewed in Katsipoulaki et al. (2024)]. Although *Candida albicans* is the more common etiological agent of infections caused by yeasts in humans, the number of infections caused by *C. glabrata* has been increasing (Katsipoulaki et al. 2024). Infections caused by *C. glabrata* are of high concern due to its low susceptibility to azoles, antifungals used prophylatically in the clinics (Salazar et al. 2020, Katsipoulaki et al. 2024, Pedro and Mira 2024). Besides this, *C. glabrata* is also capable of rapidly acquiring resistance to azoles (a reflection of its extreme genomic plasticity) resulting in decreased efficacy and worsened patient outcomes (as reviewed in Salazar et al. (2020) and Katsipoulaki et al. (2024)]. Other factors contributing for the success of *C. glabrata* as a human pathogen include its capacity to successfully colonize and adhere to a variety of biotic/abiotic surfaces (Cavalheiro and Teixeira 2018) and ability to survive inside macrophages (Seider et al. 2011, Katsipoulaki et al. 2024). Such strategy of immune evasion differs, to some extent, from the one described for *C. albicans* that relies more on escaping phagocytosis (Katsipoulaki et al. 2024). Transcriptomic profiling of *C. glabrata*-phagocytized cells revealed, among other aspects, the significant up-regulation of enzymes involved in neoglucogenesis, β-oxidation and glyoxylate cycle (Rai et al. 2012). Likely, this response reflects adaptation of the yeast cells to the portfolio of carbon sources available inside the phagosome that is depleted of glucose and enriched in fatty acids (Kaur et al. 2007, Rai et al. 2012, Fukuda et al. 2013). The inhibition of phagosome acidification, resulting in blockage of maturation and reduced activity of acidic hydrolases, was another response prompted by macrophage-engulfed *C. glabrata* cells (Seider et al. 2011).

In *C. glabrata*, the transcription factor CgHaa1 controls a large regulon of genes involved in response of this yeast to inhibitory concentrations of acetic acid at pH 4 (Bernardo et al. 2017). This same function was first described for its orthologue ScHaa1 in *Saccharomyces cerevisiae* (Mira et al. 2010b) and, later, for ZbHaa1, from the highly acetic acid-tolerant species *Zygosaccharomyces bailii* (Palma et al. 2017). Despite the similar effect in conferring protection against acetic acid, the structure of the regulatory networks controlled by ScHaa1 and CgHaa1 are considerably different, with the latter encompassing target genes (and, consequently, biological functions) not under the control of ScHaa1 (Bernardo et al. 2017). For example, CgHaa1 augmented the expression and activity of the plasma membrane proton pump *PMA1* but no similar links between control of internal pH homeostasis and the activity of ScHaa1 (or ZbHaa1) were described (Mira et al. 2010a, Bernardo et al. 2017, Palma et al. 2017). Such structural and functional differences suggest that the Haa1-dependent regulatory network evolved along the path that gave rise to *S. cerevisiae* and *C. glabrata* (Bernardo et al. 2017).

In the presence of acetic acid CgHaa1 promotes the up-regulation of nine genes predicted to encode adhesins: *CAGL0M11726g, CAGL0I11011g, CgEPA2*, Cg*AWP12*, Cg*AWP13, CAGL0H00110g, CAGL0H07469g, CAGL0F09273g*, and *CAGL0K10164g*(Bernardo et al. 2017). Using thorough genomic analyses Xu et al. (2020) revised the panoply of adhesins encoded by *C. glabrata* resulting in the reassignment of *CAGL0F09273g* to *CAGL0F09251g; CAGL0I11011g* to *CAGL0I11000g*; and in removal of *CAGL0H00110g* from the list of genes encoded by the yeast (due to RNA-seq data not corroborating expression of this gene). This study also indicated that specific features usually found in fungal adhesins were absent in *CAGL0K10164g* and *CAGL0H07469g*, leaving open the possibility that these might not be true adhesins (Xu et al. 2020). Consistent with the positive effect of CgHaa1 on the expression of the above mentioned adhesins, its deletion reduced *C. glabrata* adherence to vaginal epithelial cells while in the presence of acetic acid (Bernardo et al. 2017). A link between the activity of ScHaa1 and adhesion was also established in *S. cerevisiae* through the positive regulation of the Flo8 flocculin (Malcher et al. 2011). Adhesion/adhesiveness is a fundamental aspect of *C. glabrata* virulence whose genome encodes an exceptionally high number of adhesins, compared to *S. cerevisiae* (Weig et al. 2004, Chaudhuri et al. 2011, Xu et al. 2020). This expansion of adhesion-related genes in *C. glabrata* is hypothesized to result from a need to improve colonization of abiotic and biotic surfaces (including human epithelia) to which the cells adhere to and subsequently colonize in the form of a biofilm (Cavalheiro and Teixeira 2018). Despite their anticipated role in pathogenesis, *C. glabrata* adhesins remain poorly studied, especially compared to their *C. albicans*counter-partners (Timmermans et al. 2018). The exception is *EPA1* that received attention not only due to its involvement in pathogenesis of *C. glabrata*, but also in drug resistance (Halliwell et al. 2012, Vale-Silva et al. 2016). In this context, it is not surprising that the information available for the adhesins under the control of CgHaa1 is scarce, with only a few transcriptomic analysis demonstrating their up-regulation under oxidative stress, during growth in acidic pHs or after exposure to azoles (Roetzer et al. 2008, De Las Penas et al. 2015, Juarez-Cepeda et al. 2015, Cavalheiro et al. 2019, Goncalves et al. 2020a).

The effect of CgHaa1 in augmenting adhesion and in upregulating the expression of poorly studied adhesins prompted us to look further into the role of this regulator and of its target adhesins in *C. glabrata* adherence and colonization (in a biofilm) of biotic and abiotic surfaces. The studies were conducted under acidic conditions (pH 4) and in the presence of acetic acid, two features that have not been examined before, although the vaginal tract is acidic due to the accumulation of lactic and acetic acids produced by the commensal bacterial flora (Boskey et al. 1999). Much of the knowledge gathered about biofilm formation in *Candida* species was obtained at neutral/alkaline pHs but the molecular players underpinning formation of these structures at acidic pHs is far more elusive [as reviewed by Cavalheiro and Teixeira (2018)]. Demonstrating that pH may have a detrimental effect in shaping the molecular responses regulating biofilm formation, the Sfp1 transcription factor was demonstrated to be required for formation of *C. albicans* biofilms at acidic pHs but not at alkaline pHs (Goncalves et al. 2020b, 2023). Thus, it is essential to investigate the mechanisms involved in adherence and biofilm formation by *C. glabrata* at acidic pHs as likely they cannot be (at least not fully) extrapolated from the knowledge obtained at neutral/alkaline pHs. Herein we also implicate CgHaa1 regulatory circuit in virulence traits of *C. glabrata*, this being the first report of a biological function of this system that is not (at least, not directly) linked with acetic acid tolerance. Such identification highlights CgHaa1 as an interesting target in the development of future anti-*Candida* drugs, specially considering the alarming levels of resistance to currently used antifungals that clearly demands the identification of alternative therapeutic targets.

## Materials and methods

### Strains and growth medium

The *C. glabrata* strains used in this study are listed in Table 1. The different strains were batch-cultured at 30°C, using 250 rpm orbital agitation, in liquid YPD (Yeast Peptone Dextrose), MM (Minimal Medium), or RPMI (Roswell Park Memorial Medium) growth media. YPD contains, per liter, 20 g of glucose (Merck), 20 g bactopeptone (Difco), and 10 g yeast extract (Difco). MM medium contains, per liter, 20 g glucose (Merck), 1.7 g yeast nitrogen base without ammonium and amino acids (Difco) and 2.65 g (NH_4_)_2_SO_4_ (Merk). RPMI contains, per liter, 20 g of RPMI-1640 synthetic powder medium without glutamine (Sigma), 20 g of glucose (Merck), 0.3 g of glutamine (Sigma), and 0.165 mol/L of MOPS (Sigma). When needed, the pH of these media was adjusted to 4 using HCl as the acidulant. Solid media was obtained by supplementing the corresponding liquid medium with 20 g (per liter) of agar (Iberagar).

**Table 1.** *List of strains used in this study*.

Strain	Genotype	Predicted function of the missing encoded protein	Reference
KUE100	Parent strain derived from the *C. glabrata* strain 2001H; histidine auxotroph; the recipient enables highly efficient gene targeting in which *yku80* is repressed with a *SAT1* flipper	–	Ueno et al. (2007)
KUE100_chr606	Strain derived from KUE100 in which the *HIS3* marker was ectopically integrated at a noncoding locus of chromosome F, position 605 901–606 015	–	Ueno et al. (2011)
ΔCg*haa1*	ORF CAGL0L09339g was replaced with *CgHIS3* marker in the background of KUE100	Transcription factor, involved in the regulation of response to acetic acid stress	Bernardo et al. (2017)
ΔCg*awp12*	ORF CAGL0G10219g was replaced with Cg*HIS3* marker in the background of KUE100	Predicted cell wall adhesin	This study
ΔCg*awp13*	ORF CAGL0H10626g was replaced with Cg*HIS3* marker in the background of KUE100	Predicted cell wall adhesin	This study
Δ*CAGL0H07469g*	ORF CAGL0H07469g was replaced with Cg*HIS3* marker	Unknown, hypothesized to be involved in adhesion	This study
Δ*CAGL0K10164g*	ORF CAGL0K10164g was replaced with Cg*HIS3* marker	Unknown, hypothesized to be involved in adhesion	This study
Δ*CAGL0C03740g*	ORF CAGL0C03740g was replaced with Cg*HIS3* marker	Protein of unknown function	Bernardo et al. (2017)
Δ*CAGL0G05632g*	ORF CAGL0G05632g was replaced with *CgHIS3* marker	Protein of unknown function	Bernardo et al. (2017)
Δ*CAGL0I07249g*	ORF CAGL0I07249g was replaced with *CgHIS3* marker	Putative GTPase-activating protein involved in the cell wall and cytoskeleton homeostasis	Bernardo et al. (2017)
Δ*CAGL0K07337g*	ORF CAGL0K07337g was replaced with Cg*HIS3* marker	Predicted to play a role in the regulation of the plasma membrane ATPase activity	Bernardo et al. (2017)
ΔC*grsb1*	ORF CAGL0L10142g was replaced with Cg*HIS3* marker	Putative sphingolipid flippase	Bernardo et al. (2017)
ATCC2001	Parent strain		
ATCC2001_ Δ*Cghaa1*	Δ*Cghaa1* strain, *CgHAA1* (ORF CAGL0L09339g) was replaced by the nourseothricin resistance *SAT1* flipper cassette	Transcription factor, involved in the regulation of response to acetic acid stress	Scharwzmuller et al. (2014)

Bernardo et al. (2017) and Xu et al. (2020).

### Gene disruption

The KUE100 strain (Ueno et al. 2007) was used as the host for the individual disruption of the following CgHaa1-target genes: Cg*AWP12*, Cg*AWP13, CAGL0H07469g*, and *CAGL0K10164g*. These mutants were created by replacing their sequence with a DNA cassette containing the Cg*HIS3* gene using homologous recombination. The replacement cassette was prepared by PCR using an appropriate set of primers and using the pHIS906 plasmid containing the Cg*HIS3* sequence as a template. The transformation procedures of KUE100 cells were performed as described before (Ueno et al. 2007). The recombination locus and gene deletion were verified by PCR using appropriate primers. To obtain a wild-type strain derived from KUE100 prototrophic for histidine (as the KUE100-derived mutants), the Cg*HIS3* cassette was ectopically expressed from a noncoding locus in chromosome F resulting in strain KUE100_chr606 (Ueno et al. 2011).

### Measurement of biofilm formation in polystyrene plates by wild-type or mutant *C. glabrata* strains, in the presence or absence of acetic acid

To assess biofilms formed by the parental strain KUE100_chr606 or by the KUE100-derived mutants *ΔCghaa1*, ΔCg*awp12*, ΔCg*awp13*, ΔCAGL0H07469g, and ΔCAGL0K10164gon the surface of polystyrene plates, cell suspensions of the different strains (containing ~2 × 10^6^ cells/ml) were inoculated in 200 µl of RPMI (at pH 4) or of this medium supplemented with 30 or 45 mM acetic acid, in 96-multiwell polystyrene plates (Greiner). After inoculation, the plates were incubated, at 30°C, with orbital shaking of 100 rpm. Biofilms were sampled after 6 or 24 h after inoculation. For that, the medium was carefully removed from the wells, the remaining cells were washed twice with 100 µl PBS (Phosphate-Buffered Saline) (to remove nonadherent cells) and 100 µl of PrestoBlue (ThermoFisher Scientific) diluted solution (prepared by diluting 10X the PrestoBlue reagent in RPMI medium at pH 4) were added. Afterward, cells were incubated for 30 min at 37°C and, after that time, the absorbance at 570 nm and 600 nm was measured.

### Colonization of reconstituted vaginal epithelium by *C. glabrata* wild-type or mutant strains, in the presence or absence of acetic acid

The capability of *C. glabrata* cells of the parental strain KUE100_chr606 or of derived mutants *Δ*Cg*haa1/Δ*Cg*awp12/Δ*Cg*awp13/*ΔCAGL0H07469g*/*ΔCAGL0K10164g to adhere and colonize a commercially available reconstituted human vaginal epithelium (RHVE)(Skin Ethic 335 Laboratories; Nice, France) was assessed, exploring a previously established experimental setting (Bernardo et al. 2017). Briefly, RHVE tissues were inoculated for 12 h with 1 ml of standardized suspensions of the different *C. glabrata* strains (∼2 × 10^6^ cells/ml) in RPMI medium adjusted to pH 4, either or not supplemented with 30 mM acetic acid. As a control, two RHVE tissue preparations incubated only with 1 ml RPMI (supplemented or not with acetic acid) were also prepared. The infected and noninfected tissues were incubated, at 37°C in a 5% CO_2_ environment in saturated humidity, for 12 h. After this, the number of *C. glabrata* cells in the different tissue preparations was quantified based on quantification of the yeast genomic DNA. For this, the infected tissues were placed in sterile 1.5 ml microcentrifuge tubes (Eppendorf AG, Hamburg, Germany) with ~300 µl of glass beads (0.5 mm diameter—Sigma, St. Louis) and 600 µl of sorbitol buffer GriSP, Porto, Portugal). This final mix was homogenized three times for 60 s, using Mini-Beadbeater-8 (Stratech Scientific, Soham, UK). After tissue disruption, the supernatant was carefully removed and placed in another sterile microcentrifuge tube. Then, DNA extraction was performed using the GRS Genomic DNA kit—Tissue (GriSP), following the manufacturer’s protocol. After extraction, the DNA from each experimental condition was quantified using the NanoDrop 1000 Spectrophotometer (Thermo Fisher Scientific, Wilmington, DE). *Candida glabrata* genomic DNA was quantified by real-time PCR in a CF X96 Real-Time PCR System (Bio-Rad, Berkeley, USA) and aiming for the amplification of the *FKS2* gene. Each reaction mixture consisted of 10 µl of working concentration of SsoFast EvaGreen Supermix (Bio-Rad, Berkeley, USA), 0.2 µl of each primer (50 µM) (ATTTGCATGCGCTTGCCCACGAATCC and ACGTCTGATCCAATCAATGGCTGGTGA), and 4 µl of DNA, in a final reaction volume of 20 µl. Negative controls were performed using a reaction mixture with ddH_2_O (Cleaver Scientific Ltd, UK) substituting for the template DNA. Template DNA for each positive control was obtained from RHVE tissues after the step of DNA extraction described above. PCR cycling conditions consisted of an initial denaturation step at 98°C for 2 min, followed by 40 cycles of denaturation at 98°C for 5 s and primer annealing at 60°C for 5 s. In each cycle, a dissociation stage at 60°C was run to generate a melting curve to confirm the specificity of the amplification product. Previously, calibration curves (Ct vs. Log cells) for each *C. glabrata* strain were constructed using the same PCR protocol as described above. For these, serial dilutions of the *C. glabrata* cells were prepared and the DNA for PCR analysis was extracted from the planktonic cell pellet using the DNA extraction kit (QIAamp® DNA FFPE Tissue, Qiagen, Crawley, UK).

### Virulence of *C. glabrata* wild-type or mutant strains against the wax moth *Galleria mellonella*

To study the effect of Cg*HAA1* deletion in virulence of *C. glabrata* (in the background of the KUE100 or of the ATCC2001 strains) against the infection model *G. mellonella*, killing assays were used. Briefly, cells of the parental strain KUE100_chr606 (or of ATCC2001) and KUE100_Δ*Cghaa1* (or ATCC2011_Δ*Cghaa1*) mutant cells were cultivated, overnight, in YPD medium. On the next day, the cells were harvested by centrifugation and resuspended in an appropriate volume of PBS to yield a cellular concentration of ∼1.4 × 10^10^ cells/ml. The larvae of *G. mellonella* were raised in our laboratory from eggs to the final-instar stage on a natural diet of beeswax moth and pollen grains. They were maintained at 25°C in darkness. Final-instar larvae weighing 225 ± 25 mg were selected for the experiments. 3.5 µl of this yeast cell suspension were used to inject the larvae in the hindmost left proleg. As control, larvae were also injected with the same volume of sterile PBS. To assure the use of an identical fungal burden, serial dilutions of each suspension inoculated in the larvae were prepared and the number of colony forming units (CFUs) (formed onto the surface of solid YPD plates) quantified. At least three replicate infection assays were performed using a total of 10 larvae per assay. The survival of the larvae was recorded after 24, 48, and 72 h inoculation of the yeast cell suspension. Larvae were considered dead only if in response to shaking of the Petri dish or touch with a pipette tip the larvae displayed no movement. The same experimental setup was used to study virulence prompted by *C. glabrata* KUE100-derived strains devoid of genes Cg*AWP12*, Cg*AWP13, CAGL0H07469g, CAGL0K10164g*, Cg*BAG7, CAGL0E03740g, CAGL0G05632g, CAGL0H07469g*, Cg*HSP30*, Cg*RSB1*, and *CAGL0C03740g*.

### Interaction of wild-type and mutant *C. glabrata* strains with *G. mellonella* hemocytes

The effect of Cg*HAA1* in the ability of *C. glabrata* to interact with hemocytes obtained from the hemolymph of *G. mellonella* was determined using an adapted protocol previously established in our lab. Briefly, to isolate the hemocytes from *G. mellonella* hemolymph, the larvae (in the last-instar stage) were anesthetized on ice and after sterilization of the abdomen surface with ethanol, punctured with a sterile needle to collect the hemolymph to an anticoagulant buffer (98 mM NaOH, 145 mM NaCl, 17 mM EDTA, and 41 mM citric acid pH 4.5) in a 1:1 proportion. The hemolymph was then centrifuged at 250 g for 10 min at 4°C, washed twice with PBS, and centrifuged again at 250 g for 5 min at 4°C. The hemocytes obtained were gently suspended in 1 ml of Grace’s insect medium (GIM) (Thermo Fisher Scientific) supplemented with 10% (v/v) fetal bovine serum, 1% (w/v) glutamine, and 1% (w/v) antibiotic/antimycotic solution composed of 10 000 units of penicillin G, 10 mg of streptomycin, and 25 mg/l amphotericin B. Suspended hemocytes were counted in a hemocytometer and incubated overnight at 25°C, in 24-well plates, at a concentration of 2 × 10^5^ cell/ml. On the next day, the hemocyte monolayers were washed with PBS, and the medium was replaced with GIM without antibiotics. After this, *C. glabrata* cells of the different strains were inoculated (at a density of 7 × 10^2^ yeast cells/ml) in the hemocyte monolayers prepared. The suspension was centrifuged at 500 g for 1 min and incubated for 1 h at 37°C with 5% CO_2_. At this time the monolayer of hemocytes was washed two times with 500 µl PBS (to remove the yeast cells that were not captured by the hemocytes) and then the medium was replaced with GIM growth medium without antibiotics. After 1 and 8 h after infection of the hemocytes, 0.5% (v *v*^−1^) Triton X-100 was added to permeabilize the infected hemocytes and allow the release of the internalized yeast cells. These internalized yeast cells were quantified by preparing standardized serial dilutions of the permeabilized yeast-hemocyte co-culture followed by plating in YPD and counting the number of CFUs formed after 2 days. The same experimental setup was used to study to the interaction of *C. glabrata* mutants devoid of the CgHaa1-target genes Cg*AWP12*, Cg*AWP13, CAGL0K10164g, CAGL0I07249g, CAGL0E03740g*, and *CAGL0G05632 g* with the *Galleria mellonela* immune cells.

### Phagocytosis and intracellular killing of *C. glabrata* prompted by THP-1 human macrophage cell-line

To study the interaction of *C. glabrata* wild-type and Δ*Cghaa1* mutant cells with human immune cells we utilized the acute monocytic leukemia cell line THP-1, obtained from the American Type Culture Collection (ATCC, Rockville, MD). These cells were cultured in RPMI 1640 medium with 10% fetal calf serum and maintained at a density of 5 × 10^5^ cells/ml. THP-1 cell differentiation was induced using 50 nM of phorbol myristate acetate (PMA). After 5 days of stimulation with PMA at 37°C and with 5% CO_2_, medium was replaced with fresh RPMI medium and incubation proceeded for three more days under the same conditions. At this point, the differentiated THP-1 cells were detached from the plates by incubation, on ice, with PBS 5 mM EDTA.

To assess the capacity of the differentiated THP-1 cells to phagocytose *C. glabrata*, these cells were inoculated at a density of 1 × 10^5^ cells/well in 500 µl, in a 48-well cell plate, after which they were incubated overnight (37°C, 5% CO_2_) to allow adherence and formation of a monolayer. Counting and confirmation of viability of the THP1-cells was performed using trypan blue. On the next day, a co-culture between THP-1 and *C. glabrata* cells (KUE100_chr606 or KUE100_Δ*Cghaa1*) was established at a ratio of 1:6, corresponding to 3 × 10^7^ yeast cells/ml and 1 × 10^5^ THP-1 cells/well. The yeast cells used were obtained from a preculture made overnight in liquid YPD medium. Confirmation of the number of yeast cells present in the initial suspension was made using serial dilutions and subsequent counting of CFUs. After 1 h of the yeast/THP-1 cells co-cultivation, the supernatant was collected to remove nonphagocytosed *Candida* cells. To quantify those nonphagocytosed yeast cells, serial dilutions of the co-culture supernatant were made and the number of colonies formed onto the surface of YPD plates determined. Phagocytosis index was taken as (*C*_o_ − *C*_s_)/C_o_ × 100%, where *C*_o_ corresponds to the initial amount of yeast cells used in the co-culture and *C*_s_ is the amount of nonphagocytosed yeast cells quantified after 1 h of co-incubation with the THP1-cells.

To assess the survival capacity of *C. glabrata* while inside THP-1 cells a co-culture between yeast (KUE100_chr606 or KUE100_Δ*Cghaa1*) and the THP-1 cells was established, using the same setting as described above. After 1 h of co-cultivation, the nonphagocytosed yeasts were removed and THP-1 cells were incubated for 2–3 min in the presence of 1% TritonX-100 to promote lysis. The number of yeast cells recovered from THP-1 lysates was estimated by serial dilutions followed by counting of the number of CFUs. The intracellular killing index was calculated as (*C*_f_–*C*_MΦ_)/*C*_f_, in which *C*_f_ is the amount of ingested *C. glabrata* cells (determined as the difference between the initial number of yeast cells and the number of nonphagocytosed yeast cells, as described above) and *C*_MΦ_ is the amount of yeast cells quantified in the THP-1 lysates. The same procedure was made prior 1, 3, 6, 12, and 24 h of yeast ingestion by the macrophages to assess survival capacity of the yeasts (KUE100_chr606 or KUE100_Δ*Cghaa1*). To further detail the kinetics of killing the yeast-THP-1 co-cultures were monitored by confocal microscopy. For that, prior inoculation of *C. glabrata* cells in the co-culture these were incubated in the presence of SYTO9 (Thermo Fisher Scientific, MA). The lysosomes of THP-1 cells were stained with LysoTracker Deep Red (Thermo Fisher Scientific, MA). At designated time points THP1 cells with the ingested yeast cells were washed twice in PBS, fixed with 3.7% paraformaldehyde, washed and finally stained with DAPI (4',6-diamidino-2-phenylindole, dihydrochloride; Thermo Fisher Scientific, MA). Cells were examined under a confocal laser scanning microscopy platform TCS SP8 (Leica Microsystems, Wetzlar, Germany) equipped with 63 ×/1.40 objective (HC PL APO CS2, Leica Microsystems, Germany). Imaging was conducted with lasers at 358 nm for DAPI (λ_em_ = 461 nm), 488 nm for SYTO 9 (λ_em_ = 503 nm), and 633 nm for LysoTracker Deep Red (λ_em_ = 695 nm). For the co-localization analysis, Leica Application Suite X software (LAS X, Leica Microsystems, Wetzlar, Germany) was used. Pearson’s correlation coefficient, was calculated according to the equation:


\begin{eqnarray*}
PCC = \frac{{\sum\nolimits_i {\left( {{R_i} - \bar{R}} \right) \times \left( {{G_i} - \bar{G}} \right)} }}{{\sqrt {{{\sum\nolimits_i {{{\left( {{R_i} - \bar{R}} \right)}^2} \times \sum\nolimits_i {\left( {{G_i} - \bar{G}} \right)} } }^2}} }}
\end{eqnarray*}

where *R_i_ and G_i_* represent red and green channel intensity in each pixel, and *R^−^* and *G^−^* are red and green channel average intensity, was calculated to demonstrate positive spatial correlation. Both the phagocytosis and the killing assays were conducted in triplicates that gave rise to essentially the same results.

Statistical analyses were performed using GraphPad Prism. Data distribution was assessed using the Shapiro–Wilk test. For two-group comparisons, the Mann–Whitney U test was used. For comparisons between more than two groups, one-way ANOVA followed by Sidak’s multiple comparisons test was applied. A *P*-value < .05 was considered statistically significant.

## Results

### Role of Cg*HAA1* and of the CgHaa1-regulated genes Cg*AWP12*, Cg*AWP13, CAGL0H07469g*, and *CAGL0K10164g* in *C. glabrata* biofilm formation on abiotic and biotic surfaces in the presence of acetic acid

**Figure 1.**
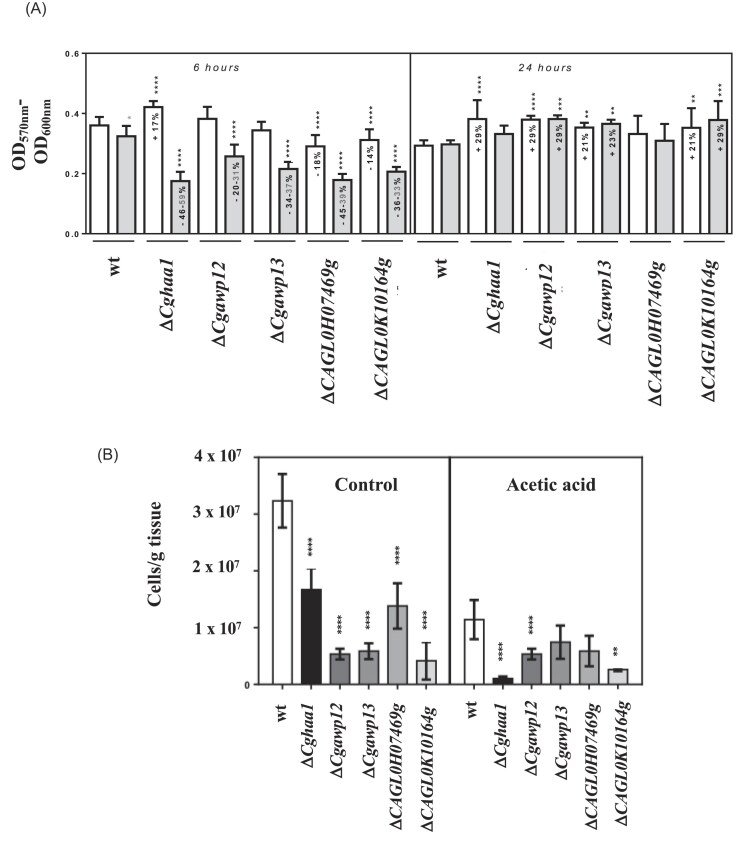
Effect of *CgHAA1* and of genes *CgAWP12, CgAWP13, CAGL0H07469g*, and *CAGL0K10164g* in *C. glabrata* adhesion and colonization (in a biofilm) of biotic and abiotic surfaces. (A) Cell viability in biofilms formed by wild-type *C. glabrata* cells (KUE100_chr606) or by the derived KUE100 mutants Δ*Cghaa1*, Δ*Cgawp12*, Δ*Cgawp13, ΔCAGL0H07469g*, and *ΔCAGL0K10164g*, after 6 or 24 h of incubation (on polystyrene plates) in RPMI (at pH4) (white bars) or in this medium supplemented with 30 mM of acetic acid (gray bars). Results represent the means of ten independent experiments and the percentages inside the bars denote the differences observed between each mutant and the wild-type. Bars without an indicated percentage correspond to those considered identical to the parental strain; (B) Number of *C. glabrata* cells recovered from the surface of vaginal cells after 12 h of co-cultivation of RVHE and Δ*Cghaa1*, Δ*Cgawp12*, Δ*Cgawp13, Δ*CAGL0H07469g, and *Δ*CAGL0K10164g cells in RPMI (at pH 4) supplemented, or not, with 30 mM acetic acid. Results represent the means of three independent experiments with the corresponding standard deviation. Statistical significance was assessed (based on a Sidak multiple comparisons test) in the co-cultures performed in the presence or absence of acetic acid, always comparing the values of the mutant strains with those obtained for the parental strain (*P < .05, **P < .01, ^****^P < .0001)

Previous work has shown that CgHaa1 increases adherence of *C. glabrata* to vaginal epithelial cells in the presence of a low concentration of acetic acid at the acidic pH of 4 (∼30 mM) (Bernardo et al. 2017). This phenotype is consistent with the transcriptional activation mediated by CgHaa1, under these same conditions, of the presumed adhesins Cg*AWP12*, Cg*AWP13, CAGL0H07469g*, and *CAGL0K10164g* (Bernardo et al. 2017). These last two genes were initially presumed to be adhesins but later found to lack essential features usually present in these proteins (Xu et al. 2020). We scrutinized the role of CgHaa1 and of its regulated adhesins in the ability of *C. glabrata* to form biofilms on the surface of abiotic (polystyrene) and biotic (reconstituted vaginal human epithelium, RHVE) surfaces, at acidic pH (pH 4) and in the presence of acetic acid (also at pH 4). For this, we compared adhesion and subsequent biofilm formation on the surface of polystyrene or of RHVE, prompted by the wild-type strain *C. glabrata* KUE100_chr606 or by the derived mutants lacking *CgHAA1, CgAWP12, CgAWP13, CAGL0H07469g*, or *CAGL0K10164g* (Fig. 1A and B). In the absence of acetic acid, along cultivation in RPMI at pH 4, only the deletion of *CAGL0H07469g* and *CAGL0K10164g*genes reduced the biofilm formed on the surface of polystyrene, although this effect was only visible at the early time point of 6 h (Fig. 1A). The supplementation of the medium with acetic acid (30 mM, pH 4) reduced, slightly, the number of adherent wild-type cells after 6 h (Fig. 1A), consistent with the low toxicity imposed on *C. glabrata* by this low concentration of acid (Lourenco et al. 2018). The acetic acid-induced reduction in early biofilm formation was more evident in all the mutants, especially for Δ*Cghaa1* where the drop reached almost 50% (Fig. 1A). The reduced biofilm formed by the mutants can result from a growth defect of the strains in the presence of the acid (resulting in a lower number of cells available to form the biofilm), from a truly reduced “adhesiveness” (capacity to adhere to the surface) or from a combination of these two aspects that are, in fact, difficult to disentangle. The increase in concentration of acetic acid to 45 mM (at the same pH of 4) led to a higher acid-induced reduction in the biofilm formed by some of the strains as visible in results shown in Supplementary Fig. S1. Under 45 mM acetic acid, the mild phenotypes of reduced biofilm formation that had been observed upon deletion of *CgAWP12* and *CgAWP13* in the presence of 30 mM acetic acid (reduction was around 35% the values observed for the wild-type strain, Fig. 1A) were no longer detectable at the early time-point of 6 h. However, a clearly reduced biofilm was observed for the strains devoid of *CgHAA1* and, less significantly, of ΔCAGL0K10164g and ΔCAGL0H07469g genes (Supplementary Fig. S1). Notably, the biofilms formed at 24 h by all the tested mutants in the presence of 30 or of 45 mM acetic acid exhibited more viable cells than those formed by the parental strain (Fig. 1A and Supplementary Fig. S1). It is possible that such differences reflect a lower maturation of the biofilms formed by these mutants, resulting from a delayed onset, compared to wild-type cells.

When the same adhesion and colonization assays were performed in the presence of the biotic surface of RHVE cultivated in RPMI at pH 4 either or not supplemented with acetic acid (30 mM), the number of Δ*Cghaa1*, Δ*Cgawp12*, Δ*Cgawp13, ΔCAGL0H07469g*, and *ΔCAGL0K10164g* cells recovered from the surface of the epithelial cells was reduced, compared to the number of cells of the corresponding parental strain (Fig. 1B). The lower colonization of the Δ*Cghaa1* mutant in the presence of acetic acid recapitulates our previous findings (Bernardo et al. 2017). All the other genes are implicated in this process of colonization of vaginal cells in the presence of acetic acid for the first time. As in the polystyrene plates, it is possible that the reduced colonization results from a reduced “adhesiveness” of the mutant strains or from a lower number of available cells to form the biofilm (due to growth defects), or from a combination of these two effects. We think the first hypothesis is more likely since we could not detect a growth defect for the strains while growing in the presence of acetic acid in a planktonic state (our unpublished results).

Two observations are of note concerning this data obtained in the presence of vaginal cells and acetic acid: (i) the lower adherence of the mutant *C. glabrata* strains to the vaginal epithelium was observed even when these cells were cultivated in the absence of acetic acid, (at a pH of 4 using HCl as the acidulant); (ii) comparing to the already acidified control, acetic acid only reduced colonization of wild-type, of Δ*Cghaa1* and *Δ*CAGL0H07469g cells (∼3-fold for the wild-type and ∼2 fold for the two mutants). These results tell us that *CgHAA1, CgAWP12, CgAWP13, CAGL0H07469g*, and *CAGL0K10164g*genes affect *C. glabrata* colonization of RHVE surface of vaginal cells more due to the acidification caused by acetic acid than to specific effects that can be specifically caused by this acid.

### Effect of CgHaa1 and of CgHaa1-regulated genes in virulence of *C. glabrata* against *G. mellonella*

**Figure 2.**
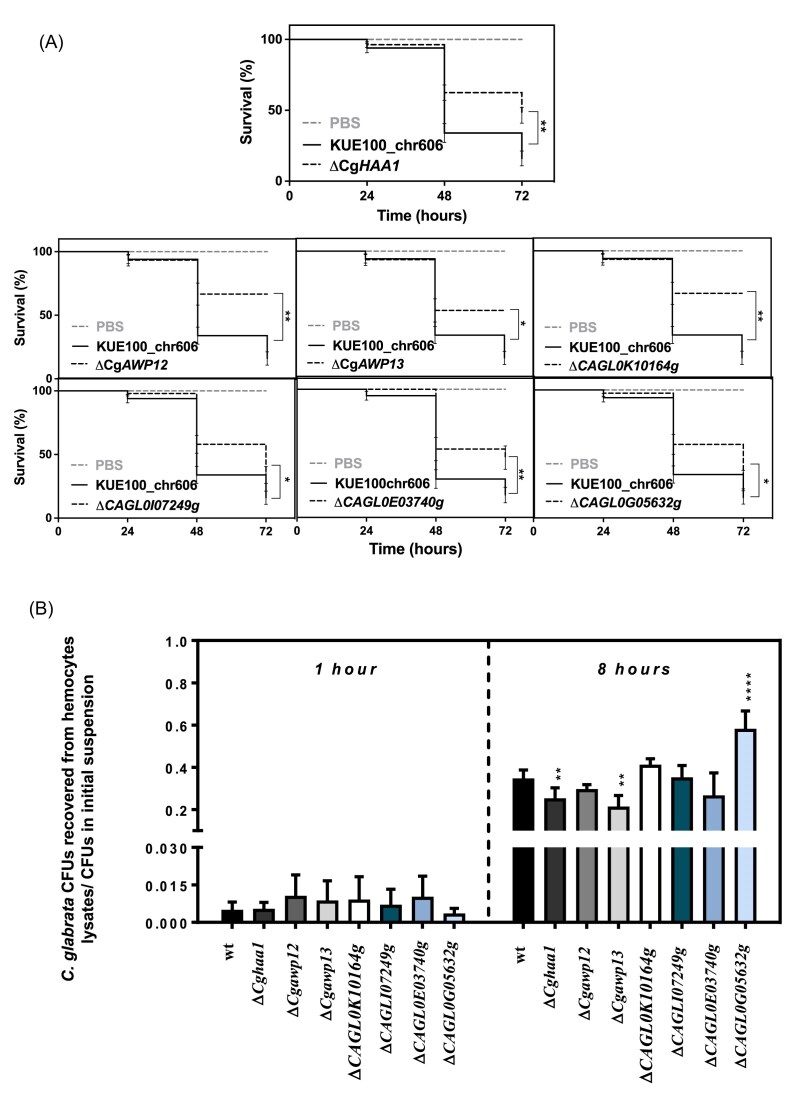
Effect of CgHaa1 and CgHaa1-target genes in killing of *G. mellonella* prompted by *C. glabrata*. (A) Survival, along 72 h, of *G. mellonella* after inoculation with *C. glabrata* wild-type (KUE100_chr606) or with the deletion mutants Δ*Cghaa1*, Δ*Cgawp12*, Δ*Cgawp13*, Δ*CAGL0K10164g*, Δ*CAGL0I07249g*, Δ*CAGL0E03740g*, and Δ*CAGL0G05632g*. Differences in survival rates were calculated by using a log-rank (Mantel-Cox) statistical test (*P* < .01, for comparison between the wild-type and each mutant); (B) Number of *C. glabrata* cells (wild-type or mutants) recovered from lysates obtained from *G. mellonela* hemocytes after 1 or 8 h of co-cultivation with the yeasts, as detailed in Materials and methods section. Statistical significance differences were assessed (using the values of the wild-type strain as a point of comparison) using a Sidak multiple comparisons test (*P < .05, **P < .01, ^****^P < .0001). All the results represent the means of at least three independent experiments.

Our results demonstrate a role for CgHaa1 and for its regulated genes in adherence and colonization of biotic and abiotic surfaces, while in the presence of acetic acid (and also during cultivation at pH 4), a key virulence trait of *C. glabrata*, specially in the context of superficial infections. Based on this, we examined the role of these proteins in pathogenesis of *C. glabrata* against the larvae of the wax moth *G. mellonella*. This wax moth has been used with success to examine the pathophysiology of *Candida* species (including of *C. glabrata*), leveraging, among other advantages, the existence of primary immune responses (Ames et al. 2017, Champion et al. 2018). We started by inoculating the wax moths larvae with an equivalent number of parental and Δ*Cghaa1* mutant cells (Fig. 2A). The *C. glabrata*-induced killing of the larvae was around 30% lower in the mutant background (Fig. 2A), a result that was also obtained in the background of ATCC2001 cells (Supplementary Fig. S2). In a second step, we inoculated the wax moths with an equivalent amount of *C. glabrata* cells obtained from Δ*Cgawp13, ΔCAGL0H07469g*, and *ΔCAGL0K10164*g mutants (Bernardo et al. 2017) (Fig. 2A). Comparing the results obtained for the parental *C. glabrata* strain, the deletion of Cg*AWP12* and *CAGL0K10164g*clearly reduced mortality of *G. mellonela*, while the deletion of Cg*AWP13* resulted in a milder, but still detectable, reduction (Fig. 2A).

To expand the analysis of CgHaa1-regulated genes to others besides those having a function related with adhesion, we decided to also monitor yeast-induced killing of *G. mellonela* in mutants devoid of *CAGL0I07249g*, encoding a predicted GTPase-activating protein involved in the cell wall and cytoskeleton homeostasis; *Cgrsb1*, encoding a predicted sphingolipid flippase; *CAGL0K07337g*, encoding a predicted regulator of the plasma membrane proton pump; *CAGL0C03740g*, encoding a predicted transcriptional regulator; and *CAGL0E03740g* and *CAGL0G5632g*, encoding genes of unknown function. All these genes were documented to be regulated by CgHaa1 under acetic acid stress (Bernardo et al. 2017) and they were chosen for these assays due to the high impact of CgHaa1 over their transcriptional regulation or because of their predicted/anticipated connection with *C. glabrata* pathogenesis. Only the deletion of the predicted GTPase activator *CAGL0I07249g* and of the unknown genes *CAGL0E03740g* and *CAGL0G5632g*reduced virulence of *C. glabrata* against *G. mellonella* (Fig. 2A).

On the next step we examined whether the reduced virulence against *G. mellonela* observed for the *C. glabrata* mutants devoid of CgHaa1 or of some of its target genes could be attributable to defects in the interaction of the yeasts with the wax moth’s hemocytes. Such aspect is relevant considering that reduced killing of the mutant strains can result from them being more efficiently killed by the wax’s immune cells. To assess this, we co-cultured (for 1 and 8 h), the different yeast strains with hemocytes obtained from *G. mellonela* lymph; after which we washed the cells, lysed the hemocytes and quantified the number of yeast cells recovered from those lysates. A first observation that emerges from these results is the higher number of yeast cells (from all strains tested) recovered from the hemocytes’ lysates obtained after 8 h of co-cultivation, compared to those recovered after only 1 h (Fig. 2B). This increase reflects a combination of factors that can include replication of the yeasts inside the hemocytes, but also the expected higher adherence and subsequent invasion of the hemocyte expected from the longer co-cultivation. A second observation of notice brought by analysis of the results is that the number of yeast cells recovered from hemocytes’ lysates after 8 h of co-cultivation was lower for the Δ*Cghaa1* and Δ*Cgawp13* (and higher for Δ*CAGL0G05632g*) mutants (Fig. 2B).

### The expression of *CgHAA1* decreases the rate of phagocytosis and subsequent killing of *C. glabrata* prompted by THP-1 macrophages

**Figure 3.**
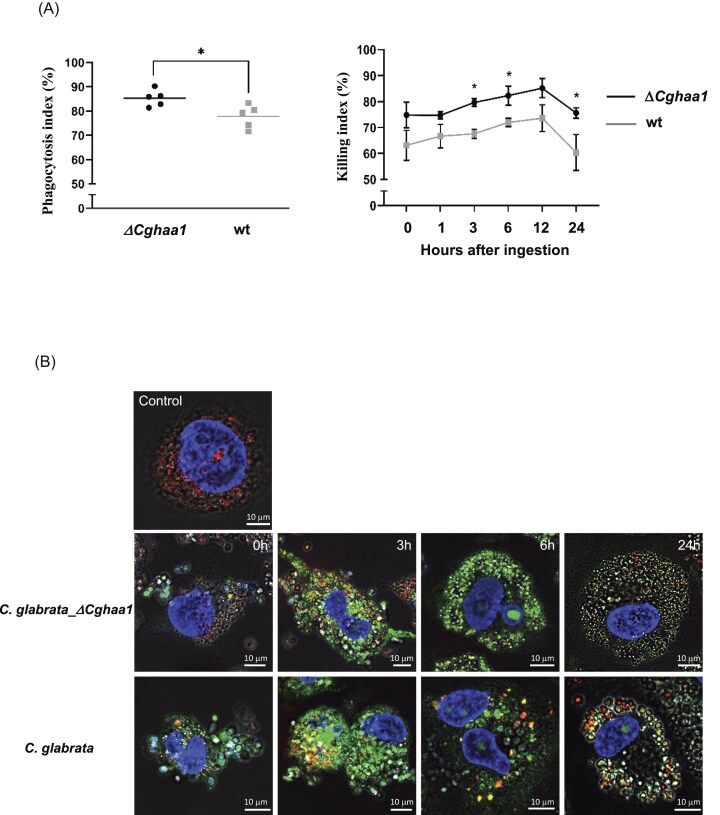

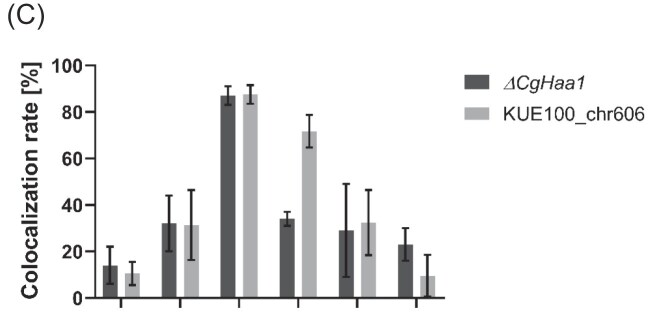
Effect of *CgHAA1* in the ability of THP-1 cells to promote phagocytosis and subsequently killing of *C. glabrata*. (A) Phagocytosis index (left) and survival inside THP-1 cells (right) of *C. glabrata* wild-type KUE100 (gray line) or Δ*Cghaa1* cells (black line). A co-culture between the yeast and the macrophage cell line was established for 1 h, after which it was calculated the percentage of ingested yeast cells (on the left). The kinetics of killing of these ingested yeast cells (from the wild-type or mutant backgrounds) was followed for 24 h, being represented the percentage of killed cells, compared to the ingested population, that was still viable (on the right). Each dot represents an individual measurement (*n* = 5 per condition); data are presented as mean ± SD. (B) To further examine the kinetics of the yeast-induced killing, fluorescence microscopy was used to accompany the co-culture, as detailed in materials and methods. Images of selected macrophage cells having ingested yeast cells of the wild-type or mutant background are exhibited. LysoTracker Deep Red (red, THP-1 lysosomes), DAPI (THP-1 blue, nuclei) and SYTO 9 (green, live *C. glabrata*) dyes were used for the imaging. Representative images are shown. (C) Colocalization rate between *C. glabrata* and lysosomes within the chase time. Data are presented as an average ± SD from no <10 cells from three independent fields of view. Statistical analysis methods are described in the Materials and methods section.

The results obtained show that CgHaa1 augments virulence of *C. glabrata* against *G. mellonela* and in part this can result from an impact of this regulator in modulating the way by which the yeasts interact with the wax moth’s immune cells. Taking this into account, we examined the interaction of wild-type and Δ*Cghaa1 C. glabrata* cells with human THP-1 macrophages. For that, we established a co-culture between THP-1 cells and the two yeast strains (wild-type and Δ*Cghaa1*) for 1 h, after which we quantified the phagocytosis index, corresponding to the percentage of ingested yeast cells compared to the population inoculated in the co-culture (Fig. 3A, left panel). Afterward, we examined the survival of those ingested yeast cells inside THP-1 macrophages over a 24-h period by quantifying viable fungal cells recovered after macrophage lysis (Fig. 3A, right panel). The deletion of *CgHAA1* clearly facilitated phagocytosis of *C. glabrata*, with the number of ingested yeast cells after 1 h of co-incubation being ~15% higher for the mutant compared to the wild-type strain (Fig. 3A, left panel). The deletion of *CgHAA1* also led to a significantly higher intracellular killing index for the mutant at 3, 6, and 24 h post-ingestion, indicating that *CgHAA1* contributes to survival of *C. glabrata* while inside the macrophages (Fig. 3A, right panel). Notably, the number of viable wild-type cells recovered after 24 h exceeded the initial number of ingested cells, resulting in a negative killing index (Fig. 3A, right panel, note the lower numbers obtained at 24 h), which suggests that *C. glabrata* can proliferate within macrophages under these conditions. This observation is consistent with previous findings (Seider et al. 2011). Colocalization analysis between yeast cells and lysosomes revealed no significant difference between the strains at 0, 1, 3, and 12 h. However, at 6 h post-ingestion, wild-type cells exhibited a significantly higher rate of colocalization with LysoTracker-positive compartments, suggesting a transient delay in lysosomal fusion in the mutant background (Fig. 3C). One interesting trend observed in the imaged cells 24 h post-ingestion, was that colocalization was higher in the mutant strain, indicating that Δ*Cghaa1* cells are ultimately more frequently routed to lysosomal compartments. This is consistent with their reduced intracellular survival and supports the notion that *CgHAA1* contributes to the evasion of degradative pathways during later stages of macrophage infection, although more firm conclusions about this will require future assays undertaken with a higher number of infected THP-1 cells. Representative fluorescence microscopy images were included to qualitatively illustrate the intracellular localization of *C. glabrata* and its spatial proximity to lysosomes within THP-1 cells (Fig. 3B).

## Discussion

The capability of *C. glabrata* to colonize a multitude of environments relies on the activity of its various adhesins that mediate differential contacts between the surface of the yeast cell and biotic or abiotic surfaces [as reviewed by Cavalheiro and Teixeira (2018) and Cavalheiro et al. (2021)]. Most of the adhesins predicted by genome analysis in *C. glabrata* remain poorly or not at all characterized (Cavalheiro and Teixeira 2018), specially at acidic pH, although this trait is relevant for vaginal pathogenesis of *Candida* as this is an acidic niche (maintained at a pH of ∼4) due to the accumulation of lactic and acetic acids (Boskey et al. 1999). Thus it is essential to establish how the presence of acetic and lactic acids (and the acidified environment their accumulation causes) impact adherence and colonization of vaginal epithelial cells by *Candida*, as well as the molecular players behind those responses. Confirming that lactic and acetic acids impact physiology of *C. glabrata*, biofilms formed by this species at pH 4 are considerably different than those formed at neutral or alkaline pHs (Goncalves et al. 2020b, 2021). Under acidic conditions a high concentration of protons will surround the yeast cells impacting the net charge of proteins and sugars protruding from the plasma membrane of the vaginal cell and from the cell wall of the yeasts, with consequences in the balance of differential contacts established. The acidic pH also increases the concentration of undissociated acetic and lactic acids that, due to their lipophilicity, will permeate the cells and, among other deleterious effects, perturb the structure of the cell wall and of the plasma membrane (Mira et al. 2010a, Cunha et al. 2017).

To avoid exclusion from the vaginal niche, *C. glabrata* cells evolved adaptive responses to cope with the presence of these organic acids at low pH including the capability to co-metabolize acetic acid and glucose and a higher control of internal pH by increased activity of the plasma membrane proton pump CgPma1 (Cunha et al. 2017). In this study, we confirmed our previous findings that the transcription factor CgHaa1 accelerates adhesion of *C. glabrata* to vaginal cells at the acidic pH of 4 and in the presence of acetic acid (Bernardo et al. 2017). We also show, for the first time, that the CgHaa1-target adhesins CgAwp12 and CgAwp13, as well as CAGL0H07469 g and CAGL0K10164g, initially predicted to be adhesins but recently found to lack essential features of this type of proteins in Fungi (Xu et al. 2020), are required for adhesion to vaginal cells when these are cultivated in the presence of acetic acid. Functional analysis of the above referred adhesins is still very scarce and thus their herein demonstrated relevance in adhesion to vaginal cells is an important step forward. Previously, adhesins Epa1, Epa6, CgPwp5 and CgAed2, were shown to mediate contacts of *C. glabrata* with vaginal cells (Mundy and Cormack 2009, Cavalheiro et al. 2021) although at nonacidic pHs and in the absence of acetic or lactic acids.

In this work, we also demonstrate, for the first time, the important role of CgHaa1 and of several of its regulated genes (the above referred adhesins but also others) in virulence of *C. glabrata* against the wax moth *G. mellonella*. Compared to other models used to study pathophysiology of *C. glabrata*, the use of *G. mellonella* is less ethically challenging and allows a tighter control of the inoculum used for the infection assay, while still offering the possibility of using a temperature of 37°C and the presence of an innate immune system comprised of a humoral and a cellular response (mediated by hemocytes capable of phagocytosis and that produce antimicrobial peptides) (Tojo et al. 2000, Trevijano-Contador and Zaragoza 2018). The expression of *CgHAA1* is required for maximal virulence of *C. glabrata* toward the nematode *C. elegans* (our unpublished results) and against human THP-1 macrophages confirming that this link with virulence is observed regardless the infection model used. The data obtained suggest that this requirement of CgHaa1 for full virulence of *C. glabrata* toward *G. mellonela* may result from an improved ability of the yeasts to cope with the larvae’s hemocytes, considering the lower number of Δ*Cghaa1* cells that could be recovered from the inside of these immune cells comparing to those recovered from the wild-type strain. Consistently, deletion of *CgHAA1* also facilitated susceptibility of *C. glabrata* to human macrophage-induced phagocytosis and subsequent killing. Interestingly, the genes transcriptionally activated in *C. glabrata* cells engulfed by human THP-1 macrophages (Rai et al. 2012) include 26% of those documented to be under CgHaa1 regulation, according with the information deposited at the PathoYeastract database (Monteiro et al. 2017). Expression of six of these described CgHaa1 targets was also found to increase killing of *G. mellonela* prompted by *C. glabrata*: Cg*AWP12*, Cg*APW13, CAGL0K10164g; CAGL0I07249g*, predicted to be involved in cell wall and cytoskeleton homeostasis; and *CAGL0C03740g* and *CAGL0G05632g*, that have unknown biological functions. Among these five targets that contribute for killing of the wax moth prompted by *C. glabrata* cells, only the expression of CgAwp13 affected the way by which the yeast cells interacted with the larvae’s hemocytes, suggesting that the way by which the other proteins contribute for killing of the larvae involves other mechanisms. As *C. glabrata* encodes several adhesins, it is possible that some functional compensation is observed in individual deletion mutants resulting in attenuated phenotypes. Since these CgHaa1 targets are still poorly functionally characterized, it is difficult to anticipate what those other mechanism(s) might be. One interesting note to account in this regard is the established link between CgHaa1 activity and the control of internal pH homeostasis in *C. glabrata* (Bernardo et al. 2017), considering that the phagolysosome is a highly acidic environment and restrain of those responses was highlighted as a relevant mechanism by which *C. glabrata* subvert macrophage activity (Seider et al. 2011).

The role of CgHaa1 in virulence of *C. glabrata* represents the first biological function described for this system (in this species but also in others where this system was described) that is not, at least directly, linked with acetic acid tolerance. Although the mechanisms controlling the activity of Haa1 had not been studied in *C. glabrata*, in *S. cerevisiae* this was found to depend on a direct binding of acetate (Kim et al. 2019) or alterations in its phosphorylation status (Sugiyama et al. 2014). The few characterizations made on the metabolites present in the hemolymph of *G. mellonella* do not report the presence of acetate (Killiny 2018) although other carboxylic acids are present (e.g. citric acid, fumaric acid or succinic acid). Whether it is a direct binding of acetate that could be eventually present inside macrophages/hemocytes (eventually resulting from metabolic activity of these cells along with some uptake from the environment) that triggers CgHaa1 activation and with that an augmentation of responses contributing for enhanced survival of *C. glabrata* inside these cells, will have to be clarified in future studies.

In conclusion, in this study, we provide evidence that the regulatory system controlled by the transcription factor CgHaa1 contribute for different aspects of *C. glabrata* virulence: promotion of the adhesion of this yeast to vaginal epithelial cells when these are exposed to acetic acid, as *in vivo*; enhanced killing of *G. mellonela* prompted by *C. glabrata*; enhanced survival inside human THP-1 macrophages. Altogether these aspects add CgHaa1-signaling system to the body of pathways contributing for pathogenesis of *C. glabrata*, a knowledge that can be used to improve the panoply of possible therapeutic targets usable in future anti-*Candida* drug-discovery studies.

## Supplementary Material

foaf054_Supplemental_File

## References

[bib1] Ames L, Duxbury S, Pawlowska Bet al. *Galleria mellonella* as a host model to study *Candida glabrata* virulence and antifungal efficacy. Virulence. 2017;8:1909–17. 10.1080/21505594.2017.1347744. 28658597 PMC5750810

[bib2] Bernardo RT, Cunha DV, Wang Cet al. The CgHaa1-regulon mediates response and tolerance to acetic acid stress in the Human pathogen *Candida glabrata*. G3. 2017;7:1–18. 10.1534/g3.116.034660. 27815348 PMC5217100

[bib3] Boskey ER, Telsch KM, Whaley KJ et al. Acid production by vaginal flora in vitro is consistent with the rate and extent of vaginal acidification. Infect Immun. 1999;67:5170–5. 10.1128/IAI.67.10.5170-5175.1999. 10496892 PMC96867

[bib4] Cavalheiro M, Costa C, Silva-Dias A et al. A transcriptomics approach to unveiling the mechanisms of in vitro evolution towards fluconazole resistance of a *Candida glabrata* clinical isolate. Antimicrob Agents Chemother. 2019;63. 10.1128/AAC.00995-18. PMC6325195 30348666

[bib5] Cavalheiro M, Pereira D, Formosa-Dague C et al. From the first touch to biofilm establishment by the human pathogen *Candida glabrata*: a genome-wide to nanoscale view. Commun Biol. 2021;4:886. 10.1038/s42003-021-02412-7. 34285314 PMC8292413

[bib6] Cavalheiro M, Teixeira MC. *Candida* biofilms: threats, challenges, and promising strategies. Front Med. 2018;5:28. 10.3389/fmed.2018.00028. PMC5816785 29487851

[bib7] Champion OL, Titball RW, Bates S. Standardization of *G. mellonella* larvae to provide reliable and reproducible results in the study of fungal pathogens. J Fungi (Basel). 2018;4:108. 30200639 10.3390/jof4030108 PMC6162639

[bib8] Chaudhuri R, Ansari FA, Raghunandanan MV et al. FungalRV: adhesin prediction and immunoinformatics portal for human fungal pathogens. BMC Genomics. 2011;12:192. 10.1186/1471-2164-12-192. 21496229 PMC3224177

[bib9] Cunha DV, Salazar SB, Lopes MM et al. Mechanistic insights underlying tolerance to acetic acid stress in vaginal *Candida glabrata* clinical isolates. Front Microbiol. 2017;8:1–13. 10.3389/fmicb.2017.00259. 28293217 PMC5329028

[bib10] De Las Penas A, Juarez-Cepeda J, Lopez-Fuentes E et al. Local and regional chromatin silencing in *Candida glabrata*: consequences for adhesion and the response to stress. FEMS Yeast Res. 2015;15:fov056. 10.1093/femsyr/fov056. 26122277

[bib11] Fukuda Y, Tsai HF, Myers TG et al. Transcriptional profiling of *Candida glabrata* during phagocytosis by neutrophils and in the infected mouse spleen. Infect Immun. 2013;81:1325–33. 10.1128/IAI.00851-12. 23403555 PMC3639592

[bib12] Goncalves B, Azevedo N, Osorio H et al. Revealing *Candida glabrata* biofilm matrix proteome: global characterization and pH response. Biochem J. 2021;478:961–74. 10.1042/BCJ20200844. 33555340

[bib13] Goncalves B, Barbosa A, Soares AR et al. Sfl1 is required for *Candida albicans* biofilm formation under acidic conditions. Biochimie. 2023;209:37–43. 10.1016/j.biochi.2023.01.011. 36669724

[bib14] Goncalves B, Bernardo R, Wang C et al. Effect of progesterone on *Candida albicans* biofilm formation under acidic conditions: a transcriptomic analysis. Int J Med Microbiol. 2020a;310:151414. 10.1016/j.ijmm.2020.151414. 32173268

[bib15] Goncalves B, Fernandes L, Henriques M et al. Environmental pH modulates biofilm formation and matrix composition in *Candida albicans* and *Candida glabrata*. Biofouling. 2020b;36:621–30. 10.1080/08927014.2020.1793963. 32674601

[bib16] Halliwell SC, Smith MC, Muston P et al. Heterogeneous expression of the virulence-related adhesin Epa1 between individual cells and strains of the pathogen *Candida glabrata*. Euk Cell. 2012;11:141–50. 10.1128/EC.05232-11. PMC3272907 22140233

[bib17] Juarez-Cepeda J, Orta-Zavalza E, Canas-Villamar I et al. The EPA2 adhesin encoding gene is responsive to oxidative stress in the opportunistic fungal pathogen *Candida glabrata*. Curr Genet. 2015;61:529–44. 10.1007/s00294-015-0473-2. 25586543

[bib18] Katsipoulaki M, Stappers MHT, Malavia-Jones D et al. *Candida albicans* and *Candida glabrata*: global priority pathogens. Microbiol Mol Biol Rev. 2024;88:e0002123. 10.1128/mmbr.00021-23. 38832801 PMC11332356

[bib19] Kaur R, Ma B, Cormack BP. A family of glycosylphosphatidylinositol-linked aspartyl proteases is required for virulence of *Candida glabrata*. Proc Natl Acad Sci USA. 2007;104:7628–33. 10.1073/pnas.0611195104. 17456602 PMC1863504

[bib20] Killiny N . Generous hosts: why the larvae of greater wax moth, *Galleria mellonella* is a perfect infectious host model?. Virulence. 2018;9:860–5. 10.1080/21505594.2018.1454172. 29726300 PMC5955462

[bib21] Kim MS, Cho KH, Park KH et al. Activation of Haa1 and War1 transcription factors by differential binding of weak acid anions in *Saccharomyces cerevisiae*. Nucleic Acids Res. 2019;47:1211–24. 10.1093/nar/gky1188. 30476185 PMC6379682

[bib22] Lourenco A, Pedro NA, Salazar SB et al. Effect of acetic acid and lactic acid at low pH in growth and azole resistance of *Candida albicans* and *Candida glabrata*. Front Microbiol. 2018;9:3265. 10.3389/fmicb.2018.03265. 30671051 PMC6331520

[bib23] Malcher M, Schladebeck S, Mosch HU. The Yak1 protein kinase lies at the center of a regulatory cascade affecting adhesive growth and stress resistance in *Saccharomyces cerevisiae*. Genetics. 2011;187:717–30. 10.1534/genetics.110.125708. 21149646 PMC3063667

[bib24] Mira NP, Becker JD, Sa-Correia I. Genomic expression program involving the Haa1p-regulon in *Saccharomyces cerevisiae* response to acetic acid. Omics. 2010a;14:587–601. 10.1089/omi.2010.0048. 20955010 PMC3125556

[bib25] Mira NP, Teixeira MC, Sá-Correia I. Adaptive response and tolerance to weak acids in *Saccharomyces cerevisiae*: a genome-wide view. Omics. 2010b;14:525–40. 10.1089/omi.2010.0072. 20955006 PMC3129613

[bib26] Monteiro PT, Pais P, Costa C et al. The PathoYeastract database: an information system for the analysis of gene and genomic transcription regulation in pathogenic yeasts. Nucleic Acids Res. 2017;45:D597–603. 10.1093/nar/gkw817. 27625390 PMC5210609

[bib27] Mundy RD, Cormack B. Expression of *Candida glabrata* adhesins after exposure to chemical preservatives. J Infect Dis. 2009;199:1891–8. 10.1086/599120. 19426114 PMC4019233

[bib28] Palma M, Dias PJ, Roque FC et al. The *Zygosaccharomyces bailii* transcription factor Haa1 is required for acetic acid and copper stress responses suggesting subfunctionalization of the ancestral bifunctional protein Haa1/Cup2. BMC Genomics. 2017;18:75. 10.1186/s12864-016-3443-2. 28086780 PMC5234253

[bib29] Pedro NA, Mira NP. A molecular view on the interference established between vaginal lactobacilli and pathogenic *Candida species*: challenges and opportunities for the development of new therapies. Microbiol Res. 2024;281:127628. 10.1016/j.micres.2024.127628. 38246122

[bib30] Rai MN, Balusu S, Gorityala N et al. Functional genomic analysis of *Candida glabrata*-macrophage interaction: role of chromatin remodeling in virulence. PLoS Pathog. 2012;8:e1002863. 10.1371/journal.ppat.1002863. 22916016 PMC3420920

[bib31] Roetzer A, Gregori C, Jennings AM et al. *Candida glabrata* environmental stress response involves *Saccharomyces cerevisiae* Msn2/4 orthologous transcription factors. Mol Microbiol. 2008;69:603–20. 10.1111/j.1365-2958.2008.06301.x. 18547390 PMC2610386

[bib32] Salazar SB, Simoes RS, Pedro NA et al. An overview on conventional and non-conventional therapeutic approaches for the treatment of candidiasis and underlying resistance mechanisms in clinical strains. J Fungi (Basel). 2020;6:23. 10.3390/jof6010023. 32050673 PMC7151124

[bib43_934_070225] Schwarzmüller T, Ma B, Hiller E et al. Systematic phenotyping of a large-scale Candida glabrata deletion collection reveals novel antifungal tolerance genes. PLoS Pathog. 2014;10:e1004211. 24945925 10.1371/journal.ppat.1004211 PMC4063973

[bib33] Seider K, Brunke S, Schild L et al. The facultative intracellular pathogen *Candida glabrata* subverts macrophage cytokine production and phagolysosome maturation. J Immunol. 2011;187:3072–86. 10.4049/jimmunol.1003730. 21849684

[bib34] Sugiyama M, Akase S-P, Nakanishi R et al. Nuclear localization of Haa1, which is linked to its phosphorylation status, mediates lactic acid tolerance in *Saccharomyces cerevisiae*. Appl Environ Microb. 2014;80:3488–95. 10.1128/AEM.04241-13. PMC4018848 24682296

[bib35] Timmermans B, De Las Penas A, Castano I et al. Adhesins in *Candida glabrata*. J Fungi (Basel). 2018;4:60. 29783771 10.3390/jof4020060 PMC6023314

[bib36] Tojo S, Naganuma F, Arakawa K et al. Involvement of both granular cells and plasmatocytes in phagocytic reactions in the greater wax moth, *Galleria mellonella*. J Insect Physiol. 2000;46:1129–35. 10.1016/S0022-1910(99)00223-1. 10817839

[bib37] Trevijano-Contador N, Zaragoza O. Immune response of *Galleria mellonella* against Human fungal pathogens. J Fungi (Basel). 2018;5:3. 30587801 10.3390/jof5010003 PMC6463112

[bib38] Ueno K, Matsumoto Y, Uno J et al. Intestinal resident yeast *Candida glabrata* requires Cyb2p-mediated lactate assimilation to adapt in mouse intestine. PLoS One. 2011;6:e24759. 10.1371/journal.pone.0024759. 21931845 PMC3170380

[bib39] Ueno K, Uno J, Nakayama H et al. Development of a highly efficient gene targeting system induced by transient repression of YKU80 expression in *Candida glabrata*. Euk Cell. 2007;6:1239–47. 10.1128/EC.00414-06. PMC1951112 17513567

[bib40] Vale-Silva LA, Moeckli B, Torelli R et al. Upregulation of the Adhesin gene EPA1 mediated by PDR1 in *Candida glabrata* leads to enhanced host colonization. mSphere. 2016;1:e00065–15. 10.1128/mSphere.00065-15. 27303714 PMC4863579

[bib41] Weig M, Jansch L, Gross U et al. Systematic identification in silico of covalently bound cell wall proteins and analysis of protein-polysaccharide linkages of the human pathogen *Candida glabrata*. Microbiology. 2004;150:3129–44. 10.1099/mic.0.27256-0. 15470094

[bib42] Xu Z, Green B, Benoit N et al. De novo genome assembly of *Candida glabrata* reveals cell wall protein complement and structure of dispersed tandem repeat arrays. Mol Microbiol. 2020;113:1209–24. 10.1111/mmi.14488. 32068314

