# Supplementary material for: The transcription factor CgHaa1 plays a role in virulence of the pathogenic yeast Candida glabrata
Source: FEMS Yeast Res. 2025 Sep 18;25:foaf054. doi: 10.1093/femsyr/foaf054 (PMC12509826; doi:10.1093/femsyr/foaf054)
Supplement: foaf054_Supplemental_File [file foaf054_supplemental_file.pdf]

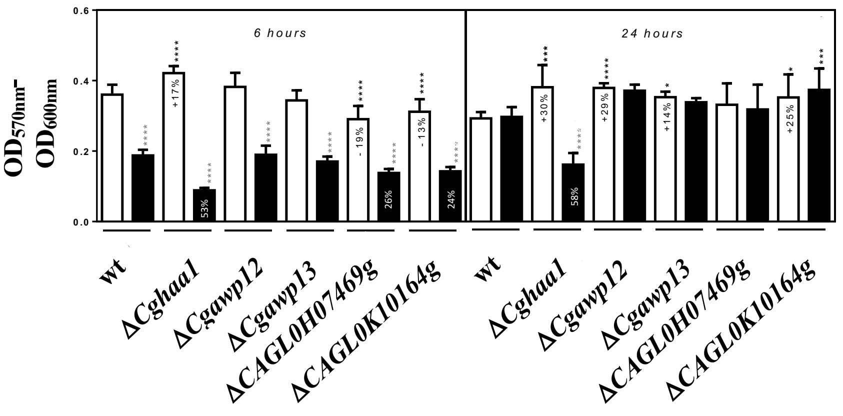


**Sup. Fig S1. Effect of *CgHAA1* and of the CgHaa1-regulated adhesins *CgAWP12*, *CgAWP13*, *CAGL0H07469g* and *CAGL0K10164g* in adhesion and biofilm formation of *C. glabrata* in biotic and abiotic surfaces.** (A) Cell viability in biofilms formed by wild-type *C. glabrata* cells (KUE100_Chr606) or by the derived mutants Δ*Cghaa1*, Δ*Cgawp12,* Δ*Cgawp13,* ΔCAGL0H07469g and ΔCAGL0K10164g, after 6 or 24 h of incubation in RPMI (at pH4), supplemented or not with 45 mM acetic acid, in polystyrene plates. Results represent the means of ten independent experiments and the percentages inside the bars denote the differences observed between each mutant and the wild-type. For the mutant strains having similar results to those of the wild-type, the percentages were not calculated. Statistical significance of the data shown was assessed (comparing to the levels obtained in the wild-type strain) using ANOVA (* $p < 0.05$, ** $p \leq 0.01$, *** $p \leq 0.01$, **** $p \leq 0.0001$).

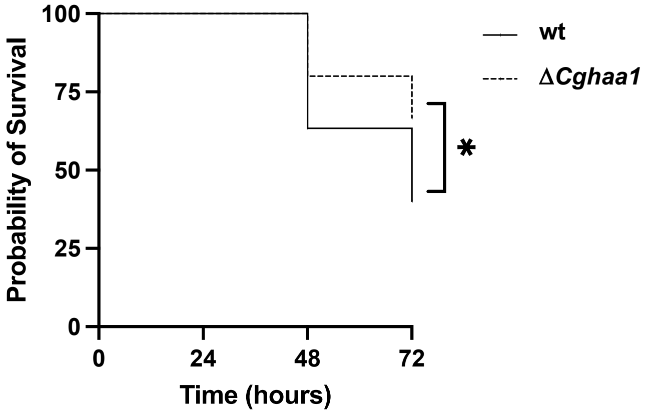


**Sup. Fig S2 Effect of CgHaa1 expression in killing of *Galleria mellonella* prompted by *C. glabrata*** (A) Survival, along 72h, of *G. mellonella* after inoculation with *C. glabrata* wild-type (ATCC2001) or with the corresponding deletion mutant ΔCghaa1. Differences in survival rates were calculated by using a log-rank (Mantel-Cox) statistical test ($p<0.01$, for comparison between the wild-type and the mutant

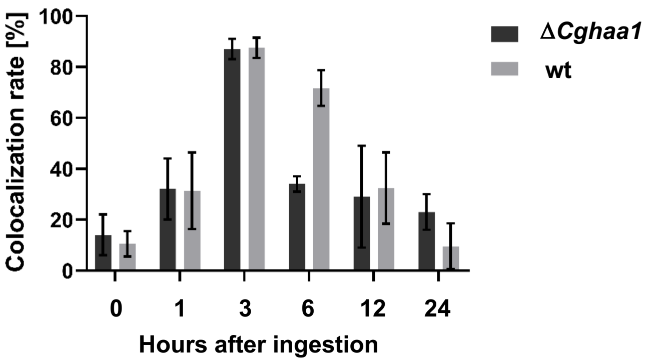


**Sup. Fig S3.** Co-localization between yeast cells and mammalian lysosomes, as determined by analysis of the images obtained by fluorescence microscopy imaging of THP-1 cells. Details on how the co-culture mas performed and subsequent steps used to accompany the fate of ingested yeast cells are provided in materials and methods.

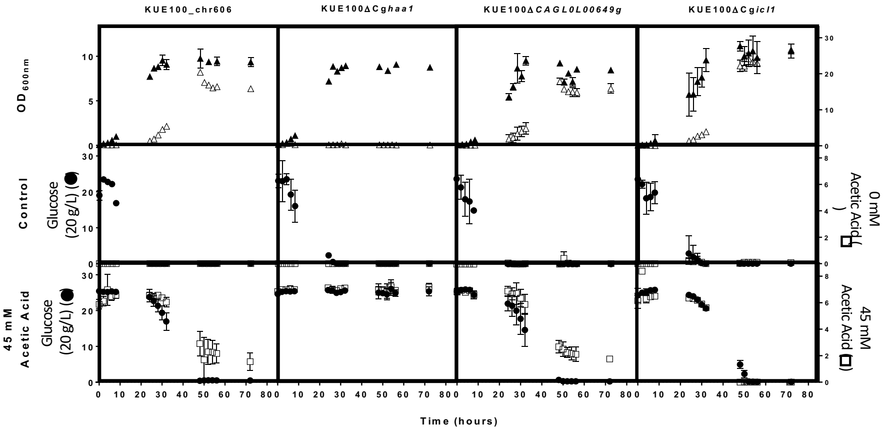


**Annex Figure 2.** *Consumption of acetic acid and glucose, the control KUE100_chr606 strain, and the deletion mutant strains ΔCAGL0L00649g* and ΔCg*icl1* were cultivated in liquid MM medium at pH 4.0 either or not supplemented with acetic acid. Growth was followed for approximately 72 h during which samples of culture supernatants were harvested and used for the quantification of acetic acid and glucose concentrations by HPLC. The results shown are means of the results obtained in three independent experiments.

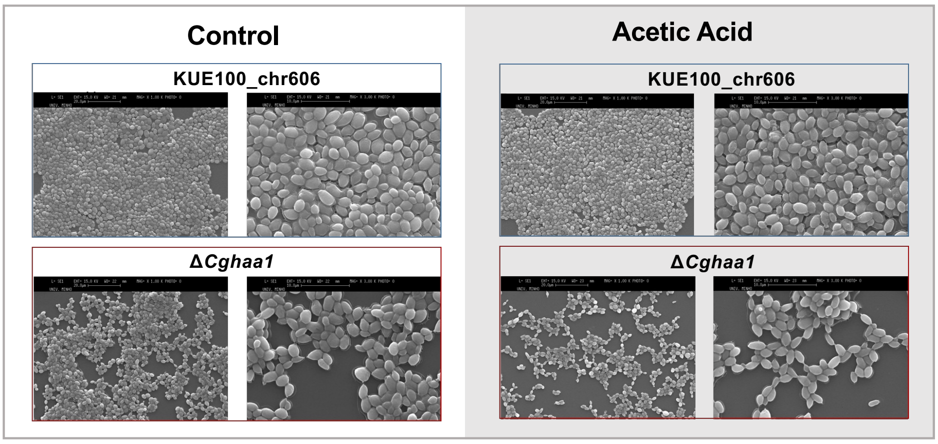


**Annex Figure 3.** *Importance of CgHaa1 in biofilm structure in vitro.* Scanning electron microscopy of *C. glabrata* control KUE100_chr606 strain and the mutant ΔCg*haa1* biofilms formed in RPMI at 24h, with or without 30mM acetic acid.
